# Evolution of benzodiazepine receptor agonist prescriptions in general practice: A registry-based study

**DOI:** 10.3389/fpubh.2022.1014734

**Published:** 2022-09-23

**Authors:** Kristien Coteur, Pavlos Mamouris, Bert Vaes, Marc Van Nuland, Catharina Matheï, Birgitte Schoenmakers

**Affiliations:** Department of Public Health and Primary Care, Academic Center for General Practice, KU Leuven, Leuven, Belgium

**Keywords:** general practice, benzodiazepines, hypnotics and sedatives, public health, inappropriate prescribing

## Abstract

**Background:**

Contrary to most European guidelines, benzodiazepine receptor agonists (BZRA) are often used continuously at a low dosage, being the most common form of long-term use. In Belgium, BZRA use is monitored by analyzing self-report data about medication use in the last 24 h. This method provides insufficient insight into the terms of use of these psychoactive drugs.

**Aim:**

To describe trends in BZRA prescribing in Flanders, Belgium, between 2000 and 2019.

**Design and setting:**

Population-based trend analysis and a case-control study for the year 2019 were done with data from a morbidity registry in general practice.

**Methods:**

Repeated cross-sectional and joinpoint regression analyses revealed trends in sex- and age-standardized prescription rates among adult patients (18+).

**Results:**

Overall, BZRA prescriptions increased. The highest overall increase was found among male patients 18–44 years old, with an average annual percentage change of 2.5 (95% CI: 0.9, 4.3). Among 65+ female patients, a decrease was found since 2006, with an annual percentage change of −0.7 (95% CI: −1.3, −0.1). In 2019, 12% of registered patients received minimally one prescription, long-term use was observed in 5%, back pain was the most common morbidity significantly associated with a rise in BZRA prescriptions, and zolpidem was the most prescribed BZRA (22%).

**Conclusion:**

Despite some statistically significant decreasing trends, an overall increase in BZRA prescriptions was observed throughout the 19-year study period, especially among long-term users of 18–44 years and 65-plus. Zolpidem became the most prescribed BZRA and warrants more attention.

## Introduction

In 2018, Belgium reported the highest consumption rate of zolpidem and the third highest consumption rate of benzodiazepines worldwide (1). With 12.73 million packs, or 434.62 million daily defined doses (DDDs) dispensed in ambulatory care, in a country with 11.38 million inhabitants in 2018, the use of benzodiazepine receptor agonists (BZRA) can be perceived as problematic (2).

BZRA are psychoactive drugs that enhance the effect of the neurotransmitter gamma-aminobutyric acid (GABA) in the central nervous system (3–9). Sedating the user, BZRA have both anxiolytic and hypnotic effects. Furthermore, they have anticonvulsive, myo- and vasorelaxant, amnestic and motor skill-impairing effects. The strength of these clinical effects is product specific. In general practice, BZRA are often used to treat insomnia, anxiety, and muscle tension, but also addiction, agitation and neurological disorders (3–5, 10–29).

The majority of BZRA in Belgium is used as hypnotics and anxiolytics, for which multiple expert groups promote a reticent policy. Belgian prescribing guidelines concur with the guidelines in most European countries, stating that BZRA should be used in the lowest possible dose and for the shortest possible duration, i.e., maximally 1–2 weeks for insomnia, and 2–4 weeks for anxiety (3, 30–33). Nonetheless, BZRA are often used continuously at a low, steady dosage, being the most common form of long-term use (12).

Although long-term use may be medically justified for some patients (34), it is associated with serious health problems, such as cognitive impairment, fall risk and resultant hip fractures, insomnia, memory disorders, especially in older populations, and drug dependence (10–18). Therefore, BZRA use should be stabilized or reduced to positively affect public health. Recently, three European registry-based studies have reported positive evolutions, namely an overall decrease in BZRA use. However, two of these studies, in Ireland and Finland, found a decrease in benzodiazepine use but an increase in z-drug use in 2006 and 2005 (34–36). In Belgium, the only published data about the evolution of BZRA use comes from patients' self-reports (37). The most recent survey results suggest a similar evolution, with a decrease in benzodiazepine users from 6.1 to 4.3% between 2004 and 2018 and a slight increase in z-drug users, from 1 to 1.2% (38). However, these results only consider patients' medication use in the last 24 h and do not provide insights into the short-term or long-term use of these psychoactive drugs. Because BZRA are only available upon prescription, the dispensed amounts suggest that patients often receive repeat prescriptions, which are provided by the general practitioner. Generally, half of the prescriptions in ambulatory care are provided by a primary care physician (39). Therefore, we aim to analyze the trends in BZRA prescription rates between 2000 and 2019 using the prescription data of the primary care-based Intego project (40).

## Materials and methods

### Intego database

Intego, which stands for “integrated computerized network”, was established by the Department of General Practice of KU Leuven in Belgium, in 1990. The database of this network contains demographic, clinical, biomedical, and prescription data, which are collected during general practitioners' daily practice. Registration with computer-generated keywords, in the electronic health record, provides a link to classifications such as the International Classification of Primary Care (ICPC-2) for diagnoses, and WHO's Anatomical Therapeutic Chemical (ATC) for medications (Supplementary overviews 1, 2). Participating general practitioners are located in the Flanders region of Belgium, where 58% of the Belgian population resides. The data was found to accurately represent the Flemish population for age and sex (40). In 2019, data was provided by 431 general practitioners, working in 86 practices with an optimal registration performance, meaning that more than 80% of their registered diagnoses were coded. The denominator was the yearly contact group (YCG), which consists of patients who visit the practice at least once in a given year (40).

The ethical committee of KU Leuven Medical School and the Belgian Privacy Commission approved the Intego procedure (ML 1723; SCSZG/13/079).

### Study design and population

A population-based trend analysis was done with data collected from 2000 to 2019. Data from the years 2020 and 2021 were excluded to prevent potential bias by COVID-19. A case-control study, in which controls were patients who did not receive BZRA prescriptions, with data from 2019, was performed to contextualize population characteristics. Patients 18 years or older who received minimally one BZRA prescription were selected.

BZRA were defined as the ATC classifications N03AE, N05BA, N05CD, and N05CF. Two groups of patients are compared: patients who received <3 BZRA prescriptions per year, and those who received three or more prescriptions in 1 year. This prescribing pattern corresponds with the most common definitions of long-term use in interventional trials, being 3–6 months of BZRA use (12, 41–53).

### Statistical analysis

A repeated cross-sectional analysis, using a Chi-square trend test with a confidence interval of 95%, was conducted for two time periods (2000 and 2019) to investigate changes in BZRA prescription rates, diagnoses, and prescriptions of other psychoactive medication. This method was also used in the case-control study. Per case, three optimally chosen controls were used. They were matched for practice, age—with a maximum difference of 2 years, and sex. These analyses were performed with R version 4.0.3 and the ccoptimalmatch R package (54, 55).

For the joinpoint regression analyses (JPRA), annual prescription rates were calculated in the total study population and in different groups: male and female, occasional and long-term users, 18–44 years, 45–64 years, and 65 years or older (65+). JPRA is a well-known method for identifying and studying statistically significant trends over time (56). The points with significant changes in prescription rates, join points, are determined by piecewise linear regression. At least four observations between two join points or three observations to the end of the data are needed to map trends. Trends are expressed by two sets of parameters: the annual percentage change (APC) and the average annual percentage change (AAPC). The APC is computed for each trend separately. Trends over the whole period of 2000–2019 were summarized using the AAPC, which is the estimated average of APC per trend weighted by the corresponding trend length. The significance of both parameters is determined with a 95% confidence interval. SEER^*^Stat package from the Surveillance Research Program of the US National Cancer Institute was used to perform JPRA (57).

## Results

### Study population

In 2000, the Intego database contained data of 79,600 patients. Of these patients, 9% (*n* = 7,209) received minimally one BZRA prescription. By 2019, this increased to 12% (*N* = 206,135; *n* = 24,962), which corresponded with a 2% rise in the sample of patients with three or more prescriptions in 1 year, and a 1% rise in the sample of patients with <3 prescriptions in 1 year (Table 1). Within both samples, the most often prescribed BZRA changed from lorazepam in 2000 to zolpidem by 2019 (Table 2).

**Table 1:** Sample characteristics based on BZRA prescription rates in 2000 and 2019.

	<**3 BZRA prescriptions**			≥**3 BZRA prescriptions**		
**Characteristic**	**2000**	**2019**		**2000**	**2019**	
	***n* = 4,690**	***n* = 14,380**		***n* = 2,519**	***n* = 10,582**	
	**%**	**%**	***p*-value**	**%**	**%**	***p*-value**
**Age**
18–44 years	27.8	28.3	0.441	13.2	11.5	0.015
45–64 years	36.9	37.6	0.367	40.0	33.1	< 0.001
65+ years	35.2	34.1	0.099	46.8	55.4	< 0.001
**Sex**
Males	36.7	38.0	0.0853	32.5	33.4	0.303
Females	63.3	61.9	0.0853	67.5	66.6	0.303
**(Co-)morbidity**
Insomnia	5.1	14.5	< 0.001	7.4	20.7	< 0.001
Anxiety	3.1	6.8	< 0.001	5.2	10.0	< 0.001
Depression	18.6	20.2	0.032	28.0	29.3	0.218
Alcohol	2.2	3.9	< 0.001	2.7	9.0	< 0.001
Psychiatric problem, other	5.5	17.1	< 0.001	7.9	20.8	< 0.001
Neurologic	7.5	8.1	0.167	10.1	12.6	< 0.001
Dementia	0.6	1.6	< 0.001	0.7	1.9	< 0.001
Hypertension	17.0	24.1	< 0.001	27.6	37.3	< 0.001
Cancer	4.8	20.8	< 0.001	6.6	25.8	< 0.001
Back pain	28.6	39.2	< 0.001	34.0	44.6	< 0.001
**Concomitant medications**
Opioids	21.2	31.3	< 0.001	30.1	45.4	< 0.001
Antidepressants	25.4	28.9	< 0.001	38.4	45.6	< 0.001
Antipsychotics	8.8	6.7	< 0.001	12.7	12.2	0.349

**Table 2:** Distribution of BZRA prescribed in 2000 and 2019 (*n*: number of prescriptions).

<**3 BZRA prescriptions**				≥**3 BZRA prescriptions**			
**2000 (*n* = 7,147)**		**2019 (*n* = 49,111)**		**2000 (*n* = 16,048)**		**2019 (*n* = 182,815)**
	**%**		**%**		**%**		**%**
Lorazepam	16.3	Zolpidem	20.7	Lorazepam	20.8	Zolpidem	22.4
Alprazolam	14.0	Alprazolam	18.2	Lormetazepam	15.6	Lormetazepam	16.9
Lormetazepam	12.3	Lorazepam	13.8	Alprazolam	13.0	Alprazolam	16.6
Zolpidem	9.8	Lormetazepam	12.2	Bromazepam	11.9	Lorazepam	14.1
Bromazepam	8.2	Diazepam	11.0	Zolpidem	6.8	Clonazepam	6.2
Other	39.4	Other	24.1	Other	31.9	Other	23.8

In 2019, 28% of patients with <3 prescriptions in 1 year and 12% of patients with three or more prescriptions in 1 year were 18–44 years old. Only in the latter sample were BZRA most prescribed to patients 65 years or older (55%). Of all patients who received a BZRA prescription, over 60% were female. In 2019, the most common diagnoses in the study population were back pain, hypertension, depression and cancer. Insomnia and anxiety held the sixth and eighth positions. All co-morbidities except depression increased significantly since 2000. Finally, a significant rise of 15% in concomitant opioid prescriptions was observed between 2000 and 2019. Among patients with three or more prescriptions in 1 year, 45% had received a prescription for opioids in 2019. A similar but less pronounced rise was also found for antidepressants, from 38 to 46% (Table 1).

### Comparison to control population

From 2000 to 2019, back pain had risen by an average factor of 1.35 in both samples (Table 1). This diagnosis was statistically associated with BZRA prescriptions when compared to the control population. Although depression did not significantly rise in the study population between 2000 and 2019 (Table 1), there was a clear association with BZRA prescriptions (Supplementary Figure S3). All diagnoses under investigation except dementia and concomitantly prescribed psychoactive drugs were significantly associated with BZRA prescribing (Supplementary Figure S3).

### Trends in BZRA prescriptions

#### Patients with <3 BZRA prescriptions in 1 year

In all age categories, a statistically significant increase was found as final trend, starting in 2012 (18–44 years: APC = 4.4; 95% CI: 2.6, 6.1; 45–64 years: APC = 2.6; 95% CI: 4.4, 3.7) and 2016 (65 years and older: APC = 4.7; 95% CI: 0.2, 9.3). In the category 18–44 years, also the overall prescription rate increased, with a significant AAPC of 1.4 (95% CI: 0.6, 2.2). Analyzing the sex-standardized trends, an overall rising trend was found in male patients, with an AAPC of 0.9 (95% CI: 0.2, 1.6). In female patients, a significant rising trend since 2012 was found in all age categories except 65 years or older. Detailed results are shown in Table 3.

**Table 3:** Trends in age- and sex-standardized BZRA prescription rates among patients who received <3 BZRA prescriptions in 1 year between 2000 and 2019.

**Group**	**2000 *N* = 7,209**	**2019** ***N* = 24,962**	**Summary**	**Trend 1**		**Trend 2**	
	**%**	**%**	**AAPC**	**Years**	**APC**	**Years**	**APC**
**Prescriptions**
**BZRA** **<** 3	65.1	57.6	0.6 (−0.1; 1.3)	2000–2019	0.6 (−0.1; 1.3)		
Overall 18–44	18.1	16.3	**1.4 (0.6; 2.2)**	2000–2012	−0.4 (−1.3; 0.6)	2012–2019	**4.4 (2.6; 6.1)**
Overall 45–64	24.1	21.7	0.4 (−0.1; 0.9)	2000–2012	–**0.9 (**–**1.5;** –**0.3)**	2012–2019	**2.6 (1.4; 3.7)**
Overall 65+	22.9	19.6	0.5 (−0.2; 1.2)	2000–2016	−0.3 (−0.7; 0.1)	2016–2019	**4.7 (0.2; 9.3)**
**Males**	23.9	21.9	**0.9 (0.2; 1.6)**	2000–2019	**0.9 (0.2; 1.6)**		
Males 18–44	7.3	6.4	**1.7 (0.8; 2.6)**	2000–2010	−0.1 (−1.6; 1.4)	2010–2019	**3.7 (2.3; 5.1)**
Males 45–64	9.2	8.6	**0.8 (0.2; 1.4)**	2000–2012	−0.6 (−1.3; 0.1)	2012–2019	**3.3 (1.9; 4.6)**
Males 65+	7.3	6.9	**0.5 (0.1; 1.0)**	2000–2019	**0.5 (0.1; 1.0)**		
**Females**	41.2	35.7	0.4 (−0.4; 1.3)	2000–2019	0.4 (−0.4; 1.3)		
Females 18–44	10.8	9.9	**1.0 (0.1; 1.9)**	2000–2012	−0.9 (−2.0; 0.2)	2012–2019	**4.3 (2.3; 6.3)**
Females 45–64	14.9	13.1	0.1 (−0.5; 0.7)	2000–2012	–**1.0 (**–**1.7;** –**0.3)**	2012–2019	**2.0 (0.7; 3.3)**
Females 65+	15.5	12.7	0.4 (−0.3; 1.2)	2000–2016	−0.3 (−0.8; 0.1)	2016–2019	4.8 (0.0; 9.8)

AAPC, average annual percentage change; APC, average percentage change; statistically significant trends (95% CI) in bold.

#### Patients with ≥3 BZRA prescriptions in 1 year

Although not all statistically significant, the latest trends in this sample were decreasing trends with APCs ranging from −4.3 (95% CI: −9.4, 1.0) to −0.7 (95% CI: −1.3, −0.1), except among female patients between 18 and 64 years. In the youngest category of female patients, an overall increase was found (AAPC = APC = 1.1; 95% CI: 0.5, 1.7). In the category of 45–64 years, trends fluctuated more (Table 4).

**Table 4:** Trends in age- and sex-standardized BZRA prescription rates among patients who received ≥3 BZRA prescriptions in 1 year between 2000 and 2019.

**Group**	**2000** ***N* = 7,209**	**2019** ***N* = 24,962**	**Summary**	**Trend 1**		**Trend 2**		**Trend 3**	
	**%**	**%**	**AAPC**	**Years**	**APC**	**Years**	**APC**	**Years**	**APC**
**Prescriptions**
**BZRA** **≥** 3	34.9	42.4	0.3 (−1.0; 1.6)	2000–2019	0.3 (−1.0; 1.6)				
Overall 18–44	4.6	4.9	**1.7 (0.6; 2.9)**	2000–2014	**3.0 (1.9; 4.0)**	2014–2019	−1.7 (−5.3; 2.1)		
Overall 45–64	14.0	14.0	0.7 (−0.3; 1.8)	2000–2004	**6.0 (0.9; 11.4)**	2004–2019	–**0.6 (**–**1.1;** –**0.1)**		
Overall 65+	16.4	23.5	**1.9 (1.1; 2.8)**	2000–2006	**8.2 (5.5; 11.0)**	2006–2019	–**0.8 (**–**1.4;** –**0.3)**		
**Males**	11.4	14.2	0.5 (−1.0; 2.0)	2000–2019	0.5 (−1.0; 2.0)				
Males 18–44	1.7	2.0	**2.5 (0.9; 4.3)**	2000–2014	**5.1 (3.6; 6.7)**	2014–2019	−4.3 (−9.4; 1.0)		
Males 45–64	4.3	4.8	**1.0 (0.1; 1.8)**	2000–2009	**3.6 (2.0; 5.2)**	2009–2019	–**1.3 (**–**2.3;** –**0.4)**		
Males 65+	5.3	7.3	**1.6 (0.8; 2.3)**	2000–2007	**6.4 (4.4; 8.6)**	2007–2019	–**1.2 (**–**1.8;** –**0.6)**		
**Females**	23.6	28.2	0.5 (−1.3; 2.4)	2000–2019	0.5 (−1.3; 2.4)				
Females 18–44	2.9	2.9	**1.1 (0.5; 1.7)**	2000–2019	**1.1 (0.5; 1.7)**				
Females 45–64	9.7	9.2	0.2 (−1.3; 1.8)	2000–2008	**2.3 (0.5; 4.0)**	2008–2012	−4.5 (−10.6; 2.0)	2012–2019	0.7(−1.0; 2.4)
Females 65+	11.0	16.1	**2.1 (1.2; 3.0)**	2000–2006	**8.5 (5.6; 11.6)**	2006–2019	–**0.7 (**–**1.3;** –**0.1)**		

AAPC, average annual percentage change; APC, average percentage change; statistically significant trends (95% CI) in bold.

In all categories, significant increases were found before any decreasing trends, resulting in overall rising trends with significant AAPCs ranging from 1.0 (95% CI: 0.1, 1.8) to 2.5 (95% CI: 0.9, 4.3) (Table 4). The fluctuations and strength of these trends are illustrated in Supplementary Figures S4.4–S4.6.

## Discussion

### Key findings

Inappropriate BZRA prescribing, at odds with current guidelines, seems to be highly prevalent in Belgium. First, throughout the 19-year study period there was an increase in patients receiving three or more BZRA prescriptions in 1 year, particularly among male patients 18–44 years old and female patients 65 years or older, despite some significant decreasing trends. Second, back pain was the most common diagnosis associated with BZRA prescribing, even though Belgian guidelines recommend against the use of muscle relaxants (58). Diagnoses of anxiety and insomnia, two of the main indications for BZRA use, were rather limited in the study population (on average 8% anxiety and 17.6% insomnia). Nevertheless, the hypnotic zolpidem was the most prescribed BZRA in 2019, accounting for 22% of all registered BZRA prescriptions. Finally, comparing characteristics to a matched control sample showed that all diagnoses, except dementia and concomitantly prescribed psychoactive medication, were significantly associated with BZRA prescription.

### Context

In comparison to registry-based data from 2014 to 2015, long-term BZRA use, approximated by receiving three or more BZRA prescriptions in 1 year, is one to two percent more prevalent in Belgium than in European countries such as France and Finland (34, 59). Consistent with previous reports of increased trends in zolpidem use (34, 36, 38), zolpidem became the most prescribed BZRA in Belgium by 2019. This could be due to professionals' perception of z-drugs as more beneficial than benzodiazepines. Their clinical experience with z-drugs overemphasizes the effectiveness in treating insomnia. Moreover, care professionals believe that patients experience significantly fewer side effects when using z-drugs over benzodiazepines (60–62).

Although previous studies have shown that long-term BZRA use is most commonly related to various psychiatric conditions, such as anxiety and depression (12, 35, 63), the most common diagnosis in our study population was back pain. This was followed by hypertension, which was also highly prevalent in the cohort that Torres-Bondia et al. studied (35). Insomnia and anxiety held the sixth and eighth positions, leading us to hypothesize that they are not consistently coded as a diagnosis when being secondary to a somatic disease. This was also suggested by Rosman et al., who found no correlation between the effect of somatic disease diagnoses, and insomnia and anxiety diagnoses, on BZRA prescribing (64). Conversely, BZRA have both myorelaxant and vasodilatory effects so it cannot be ruled out that they are sometimes prescribed for treating the aforementioned conditions.

Finally, in the youngest age category, 18–44 years, all significant findings are rising trends, with an overall increase (AAPC = 1.7; CI: 0.6, 2.9) among patients who received three or more prescriptions in 1 year. Moreover, in 2018, Sidorchuck et al. reported that in Sweden 31% of all 18–24 years old BZRA users received prescriptions to use this medication for more than 6 months (63). These findings highlight the risk of inappropriate long-term prescribing continuing in future generations, as previously described by Cadogan et al. (36).

### Strengths and limitations

To our knowledge, this is the first registry-based research that covers a study period of 19 years to describe BZRA prescription trends in primary care. Moreover, comparing trends between two groups of patients, differentiating long-term use from other use, was discussed in only one other recent study (34). Furthermore, we used data from a large real-world study population, representative of the general Flemish population in terms of age and sex (40). Although this is a major strength, this method also brings a few limitations. Data from the Intego project includes coded diagnoses and medication prescriptions, extracted from the electronic health record in general practice. Paper prescriptions, prescriptions by specialists, the dosage, and the frequency of use by the patient were not available. Additionally, data could be influenced by evolutions in coding practices, related to the further development of electronic health record systems and electronic prescribing, or the quality of registration by the general practitioner. Although this may result in an underestimation of prescription rates, it is not expected to affect the direction of the reported trends. Another limitation lies with the exclusion of the data from 2020 to 2021 to prevent potential bias by COVID-19. Although it would be interesting to study the prescribing of hypnotics and anxiolytics during the COVID-19 pandemic, we aimed to map general BZRA prescription trends in primary care. Moreover, an analysis of BZRA prescription trends during COVID-19 would be more interesting in a few years, as we hypothesize that sleep disturbances and anxiety will stabilize to pre-pandemic levels because of patients' resilience.

Finally, during analysis it was difficult to compare the observed prescribing rates with reports from other countries because of differences in the origin of the datasets, patient populations, definition of long-term use, and time periods. However, in their systematic review, Kurko et al. suggest defining long-term use as at least 6 months' use or longer during 1 year, for future research (12). On the one hand, we concur with them and plead for standardization. On the other hand, we did not adhere to this criterion in the current project because the dosage prescribed was not available. Analysis was based on the medication code, patient identifier, and date of prescription. Therefore, it is possible that some prescriptions allowed patients to buy medication for a longer period of time, as is regularly observed in clinical practice when prescribing to chronic users. Therefore, we opted for the minimal threshold of three prescriptions, hypothesizing that this corresponds to minimally 3 months of use. For future projects, we will be able to consider the dosage prescribed by the GP because of a recent update of the Intego database. This will contribute to constructing a more precise proxy for the concept of long-term use.

### Implications for research and practice

To improve (de)prescribing practices, the BZRA situation in Belgium demands both interventional and epidemiological studies. First, implementation research to increase non-pharmacological treatment and discontinuation interventions is required. This could be linked to mapping the patients' access to mental health services and accessibility of care. Future interventions could also focus more on empowering patients to discuss their medication use and possible non-pharmacological treatment options. Moreover, policy and guidelines should motivate general practitioners to discontinue long-term BZRA use that is no longer medically justified. Tools that help them regularly review their prescribing practices could be useful in this matter. Second, the significant prevalence of back pain and concomitant opioid prescribing in a BZRA-consuming population warrants further research. Since both BZRA and opioids have sedating effects for which tolerance is rather quickly developed, the impact of concomitantly prescribing them in this population should be investigated. Third, the Belgian guidelines on the treatment of insomnia recommend a maximum of 1 week of pharmacological treatment, yet 22% of all BZRA prescriptions in the group that received three or more prescriptions per year are for zolpidem, a hypnotic drug. Professionals' attitudes toward z-drugs (60–62) may lead to an inadequate judgment of these drugs' risk-benefit ratio. Since they cause the same adverse side effects as benzodiazepines when used in the long term, including their potential for recreational abuse (65), future campaigns about BZRA discontinuation should explicitly mention and possibly target z-drugs prescribing behavior. Finally, when comparing to a matched control population, all diagnoses except dementia, and concomitant prescriptions of psychoactive drugs were significantly more prevalent in our study population. Further research could clarify whether this comes from the complexity and multimorbidity in BZRA-consuming patients, or inappropriate prescribing or outdated coding.

## Data availability statement

The original contributions presented in the study are included in the article/Supplementary material, further inquiries can be directed to the corresponding author.

## Author contributions

CM conceived the study. PM prepared the dataset and performed the cross-sectional and case-control analyses. KC performed the joinpoint regression analysis with guidance of PM. KC wrote the first draft of the article. All authors contributed to the study design and approved the final version of the article.

## Conflict of interest

The authors declare that the research was conducted in the absence of any commercial or financial relationships that could be construed as a potential conflict of interest.

## Publisher's note

All claims expressed in this article are solely those of the authors and do not necessarily represent those of their affiliated organizations, or those of the publisher, the editors and the reviewers. Any product that may be evaluated in this article, or claim that may be made by its manufacturer, is not guaranteed or endorsed by the publisher.
