# Erratum: Evolution of benzodiazepine receptor agonist prescriptions in general practice: A registry-based study

**DOI:** 10.3389/fpubh.2022.1107376

**Published:** 2022-12-13

**Authors:** Frontiers Production Office

**Affiliations:** Frontiers Media SA, Lausanne, Switzerland

**Keywords:** general practice, benzodiazepines, hypnotics and sedatives, public health, inappropriate prescribing

Due to a production error, there was a mistake in the legend of **Table 4** as published. Both **Tables 3** and **4** were published with the same captions. The correct legend of **Table 4** appears below.

**Table 4**. Trends in age- and sex-standardized BZRA prescription rates among patients who received ≥3 BZRA prescriptions in 1 year between 2000 and 2019.

Due to a production error, the title of a sub-section was published incorrectly.

A correction has been made to the section **Results**, subsection **Trends in BZRA prescriptions**, paragraph 2. The correct title appears below:

“Patients with ≥3 BZRA prescriptions in 1 year.”

The publisher apologizes for these mistakes. The original article has been updated.

