# Supplementary material for: Evolution of benzodiazepine receptor agonist prescriptions in general practice: A registry-based study
Source: Front Public Health. 2022 Sep 23;10:1014734. doi: 10.3389/fpubh.2022.1014734 (PMC9546292; doi:10.3389/fpubh.2022.1014734)
Supplement: Supplementary file 1 [file Data_Sheet_1.docx]

Supplementary Material

# Overview of morbidities related to the clinical effects of benzodiazepine receptor agonists (BZRA)

| **Morbidity** | **ICPC-2 codes** |
| --- | --- |
| Insomnia | P06 |
| Anxiety | P01, P74 (P74.01, P74.02), P75, P79, P80 (P80.01) |
| Depression | P03, P76 (P76.02) |
| Alcohol | P15.01, P15.05 |
| Psychiatric problem, other | P18, P29, P71 (P71.04), P73 (P73.02), P79.01, P98, P99 (P99.01) |
| Neurologic | F14, K90, N01, N02, N06.01, N04, N86, N87, N88, N90, N99 (N99.01, N99.02, N99.03) |
| Dementia | P70 (P70.01, P70.02) |
| Hypertension | K82, K86, K87 |
| Cancer | B74 (B74.01), D74, D75, D76, D77, L71 (L71.01, L71.02), U75, U76, U77, R84, R85, S77.01, T71, X76, X77 (X77.01, X77.02), Y77, Y78.03 |
| Back pain | L02, L03 |

# Overview of ATC-codes in the category BZRA, opioids, antidepressants, and antipsychotics

Benzodiazepine receptor agonists

N03AE: antiepileptic benzodiazepine derivatives

N05BA: anxiolytic benzodiazepine derivatives

N05CD: hypnotic and sedative benzodiazepine derivatives

N05CF: hypnotic and sedative benzodiazepine related drugs

Opioids

N02: analgesics

Antidepressants

N06AA: non-selective monoamine reuptake inhibitors

N06AB: selective serotonin reuptake inhibitors

N06AF: monoamine oxidase inhibitors, non-selective

N06AG: monoamine oxidase A inhibitors

N06AX: other antidepressants

N06CA01: amitriptyline and psycholeptics

N06CA02: melitracen and psycholeptics

N06CA03: fluoxetine and psycholeptics

Antipsychotics

N05A: antipsychotics

# Results of matched case-control study

Table S3.1. Matched case-control study for patients in 2019 (3 controls per case; controls can be present in both control groups if they are an exact match to the case for age, sex, and GP)

|  | < 3 BZRA prescriptions | | | ≥ 3 BZRA prescriptions | | |
| --- | --- | --- | --- | --- | --- | --- |
| Characteristic | **case**  **n=13,940** | **control**  **n=41,661** |  | **case**  **n=10,217** | **control**  **n=30,040** |  |
|  | % | % | p-value | % | % | p-value |
| Age |  |  |  |  |  |  |
| 18-44 years | 28.4 | 28.5 | 0 | 11.4 | 11.6 | 0 |
| 45-64 years | 37.8 | 37.8 | 0 | 33.1 | 33.8 | 0 |
| 65+ years | 33.8 | 33.6 | 0 | 55.4 | 54.6 | 0 |
| Sex |  |  |  |  |  |  |
| Males | 38.1 | 38.2 | 0 | 33.6 | 34.1 | 0 |
| Females | 61.9 | 61.8 | 0 | 66.4 | 65.9 | 0 |
| (Co-)morbidity |  |  |  |  |  |  |
| Insomnia | 14.6 | 6.2 | <0.001 | 20.9 | 6.4 | <0.001 |
| Anxiety | 6.8 | 2.7 | <0.001 | 9.8 | 2.6 | <0.001 |
| Depression | 20.0 | 10.1 | <0.001 | 29.3 | 10.3 | <0.001 |
| Alcohol-drugs | 3.9 | 1.7 | <0.001 | 9.1 | 1.8 | <0.001 |
| Psychiatric problem, other | 17.2 | 9.5 | <0.001 | 20.8 | 8.7 | <0.001 |
| Neurologic | 8.1 | 5.7 | <0.001 | 12.5 | 6.9 | <0.001 |
| Dementia | 1.5 | 1.4 | 0.700 | 1.9 | 2.1 | 0.290 |
| Hypertension | 24.0 | 21.9 | <0.001 | 37.4 | 30.0 | <0.001 |
| Cancer | 20.9 | 18.2 | <0.001 | 25.9 | 21.2 | <0.001 |
| Back pain | 39.4 | 32.2 | <0.001 | 44.5 | 33.6 | <0.001 |
| Concomitant medications |  |  |  |  |  |  |
| Opioids | 31.3 | 17.7 | <0.001 | 45.2 | 19.0 | <0.001 |
| Antidepressants | 28.7 | 9.8 | <0.001 | 45.4 | 10.4 | <0.001 |
| Antipsychotics | 6.6 | 2.2 | <0.001 | 12.0 | 2.4 | <0.001 |


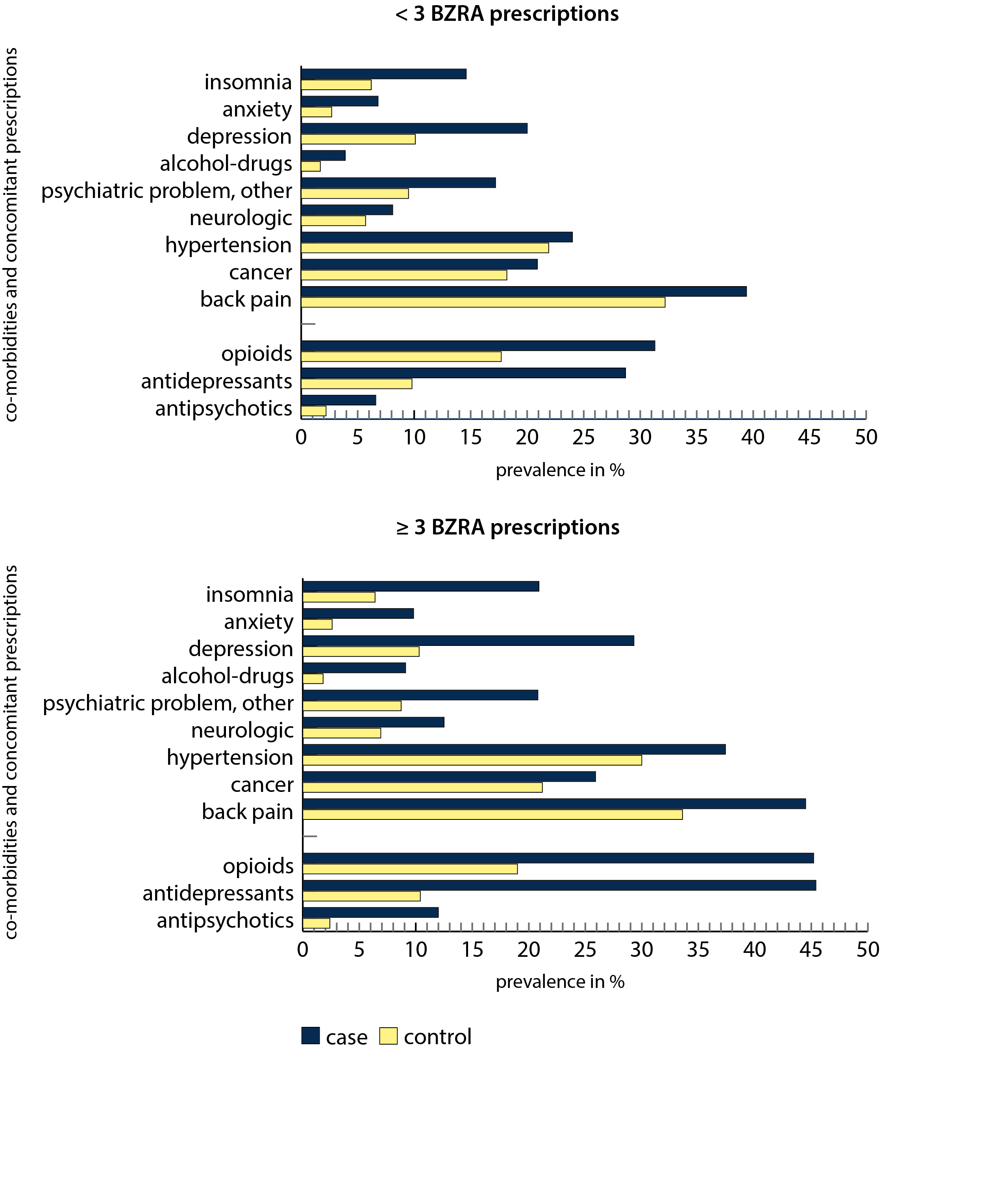


Supplementary figure S3.1. Co-morbidities and concomitant prescriptions with statistically significant differences (p-value of <0.001) in prevalence (%) between matched cases and controls in 2019. Detailed results can be found in Table S3.1.

# Visual presentation of joinpoint regression analyses results

**
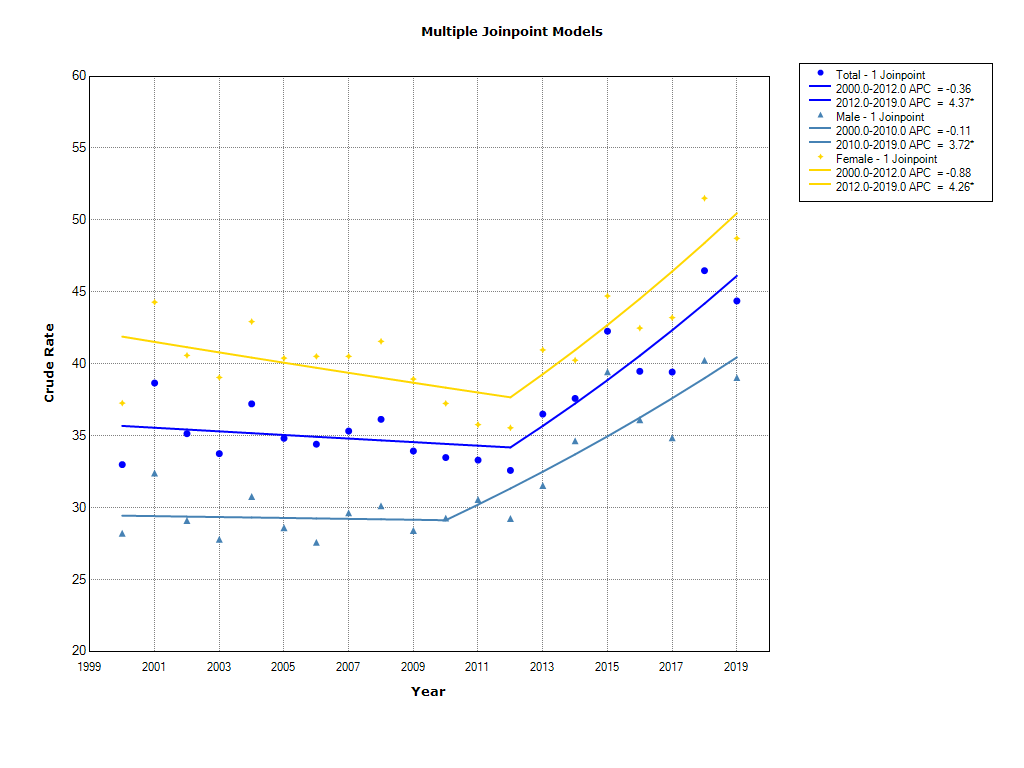
**

Supplementary figure S4.1. Patients with less than three BZRA prescriptions in one year of 18 to 44 years old

**
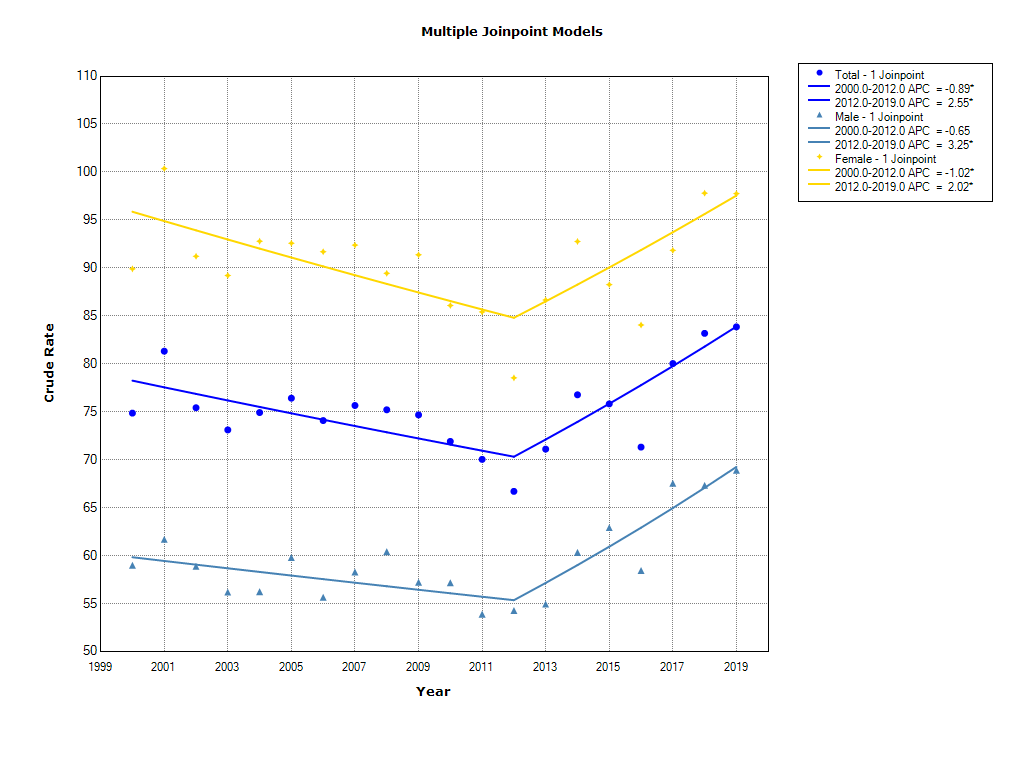
**

Supplementary figure S4.2. Patients with less than three BZRA prescriptions in one year of 45 to 64 years old

**
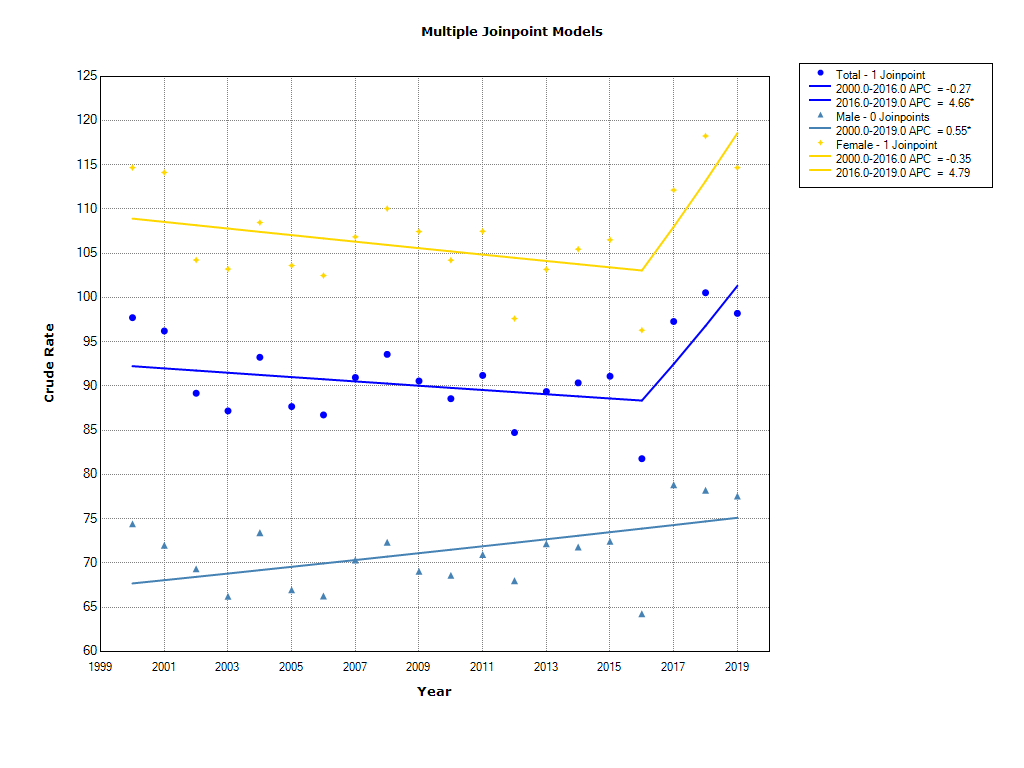
**

Supplementary figure S4.3. Patients with less than three BZRA prescriptions in one year of 65 years or older

**
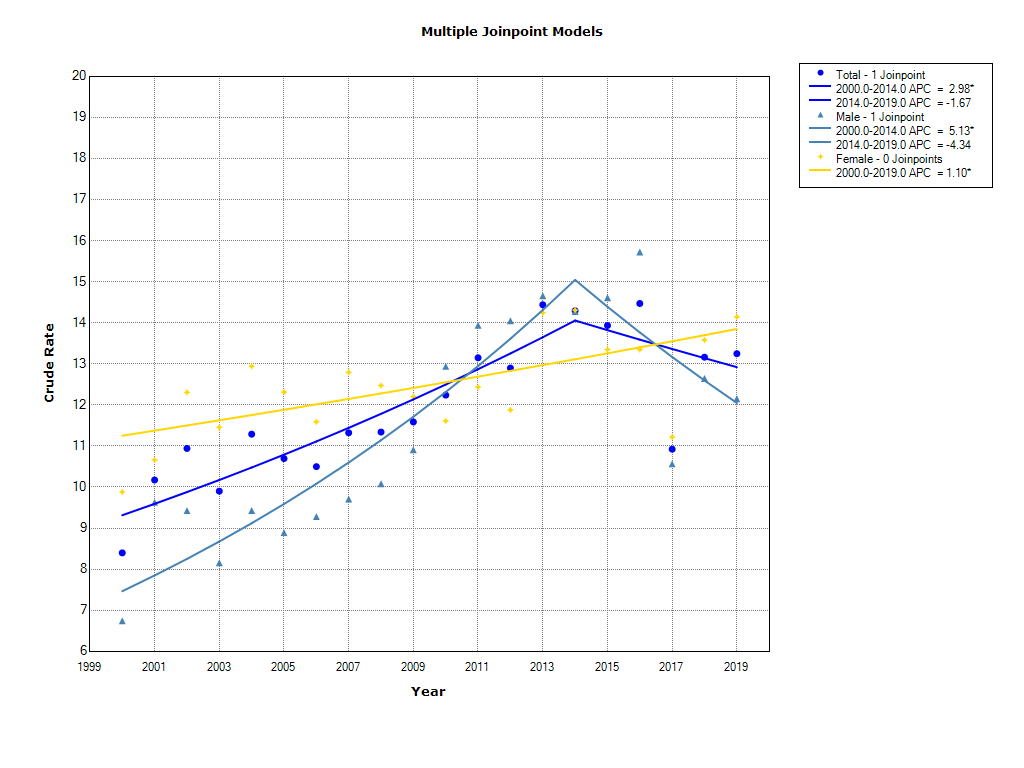
**

Supplementary figure S4.4. Patients with three or more BZRA prescriptions in one year of 18 to 44 years old

**
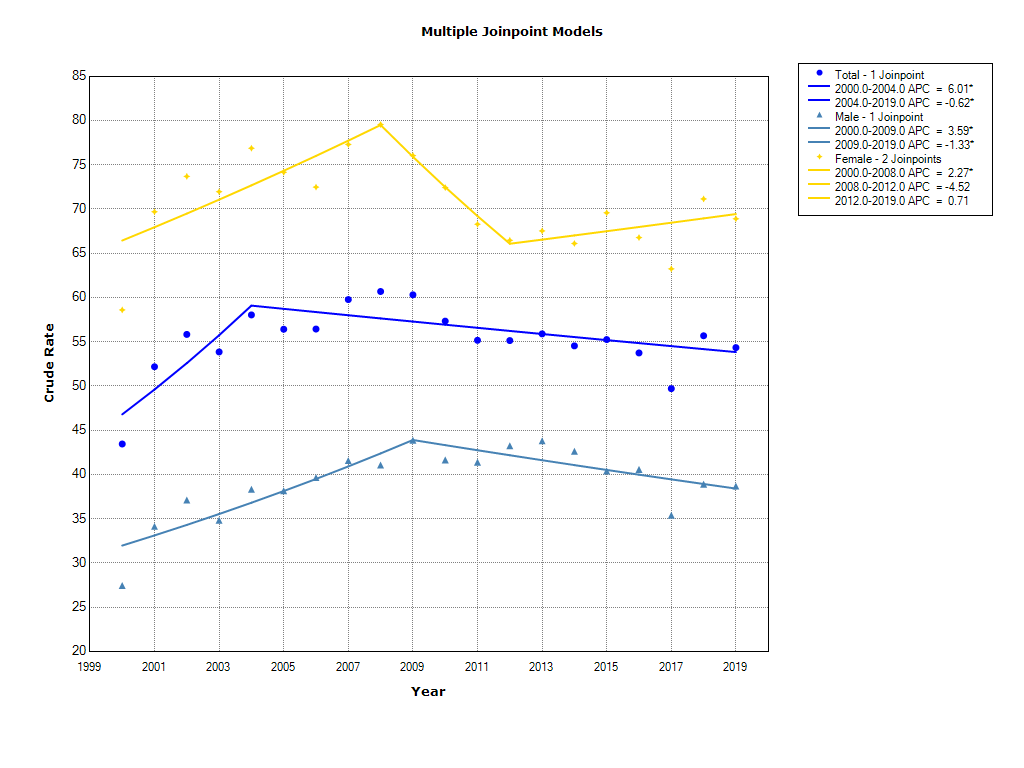
**

Supplementary figure S4.5. Patients with three or more BZRA prescriptions in one year of 45 to 64 years old


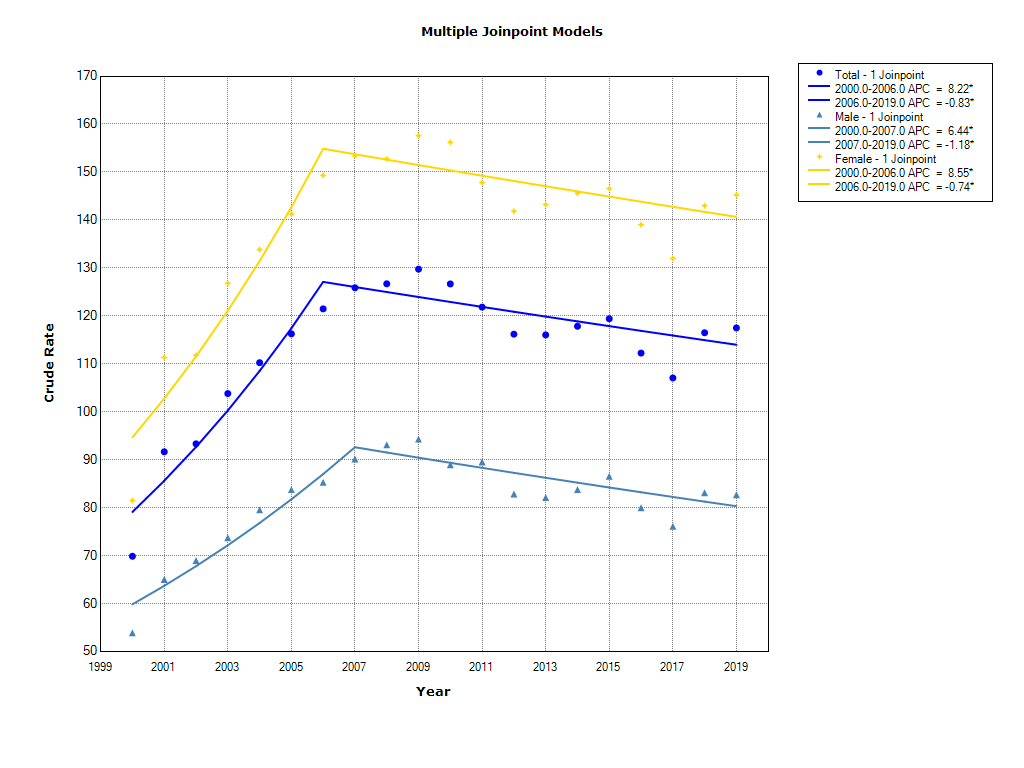


Supplementary figure S4.6. Patients with three or more BZRA prescriptions in one year of 65 years or older
